# Study on the CO Oxidation over Ceria-Based Nanocatalysts

**DOI:** 10.1186/s11671-016-1375-z

**Published:** 2016-03-24

**Authors:** Marco Piumetti, Tahrizi Andana, Samir Bensaid, Nunzio Russo, Debora Fino, Raffaele Pirone

**Affiliations:** Department of Applied Science and Technology, Politecnico di Torino, Corso Duca degli Abruzzi 24, 10129 Torino, Italy

**Keywords:** CO oxidation, Properties of cerium-based oxides, Nanostructured ceria, Ceria-based catalysts, Structure sensitivity

## Abstract

A series of ceria nanocatalysts have been prepared to study the structure dependency of the CO oxidation reaction. The ceria samples with well-defined nanostructures (nanocubes/Ce-NC and nanorods/Ce-NR) have been prepared using the hydrothermal method. Mesoporous ceria (Ce-MES) and ceria synthesized with solution combustion technique (Ce-SCS) have also been prepared for comparison. The lowest CO oxidation temperature has been reached by using ceria nanocubes (Ce-NC). This high activity draws immense contributions from the highly reactive (100) and (110) surfaces of the truncated nanocubes. The Ce-MES and Ce-SCS samples, despite their high surface areas, are unable to outdo the activity of Ce-NC and Ce-NR due to the abundant presence of (111) crystalline planes. This finding confirms the structure sensitivity of CO oxidation reaction catalyzed with ceria.

## Background

During the last few decades, ceria-based materials have been widely used for many catalytic applications, including diesel soot combustion, CO oxidation, and VOC abatement [1–4]. These oxidation processes take advantage of the unique redox properties and high oxygen storage capacity (OCS) of ceria. In particular, CO oxidation, a primary function of three-way catalytic converters (TWCs), diesel oxidation catalysts (DOC), and diesel particulate filters (DPF), can be a prototypical reaction for probing the oxidation activity of ceria-based materials [5–8]. As a result, many studies have been carried out to evaluate the catalytic oxidation of CO by ceria and related materials. According to the literature [1, 5, 8, 9], CO oxidation over ceria-based catalysts takes place via a Mars-van Krevelen (MvK)-type mechanism, whereby the reaction involves alternating reduction-oxidation steps on the solid surface with the formation of oxygen vacancies and their successive replenishment by gas-phase oxygen. However, a general consensus has been reached on the fact that CO interaction with ceria is structure sensitive [5, 8]. In addition, it has been revealed that ceria nanoparticles (i.e., nanowires and nanorods), exhibiting an abundance of (110) and (100) crystalline planes, are catalytically more active toward several reactions than CeO_2_ particles with preferred exposure of stable (111) facets [8, 10–15].

Exposure of specific crystallographic facets, together with the increased number of edges, corners, and facets, is pivotal in controlling the surface reactivity. In fact, nanocatalysts with multifaceted morphologies are highly desirable in catalysis. For fcc metal nanoparticles, the surface energy density (*γ*) for the three lowest index planes can be ordered as *γ*_(111)_ < *γ*_(100)_ < *γ*_(110)_ [16, 27]. Experimental studies have confirmed that this trend may also hold for ceria-based materials [16, 17]. Theoretical work on the interaction among CO and CeO_2_ planes have shown the structure sensitivity for CO adsorption, thus suggesting weak CO adsorption for the most stable (111) surface and stronger chemisorption with (100) and (110) facets [18–23]. In this scenario, the metastable ceria (110) surface seems to be the most promising candidate for CO oxidation, since the formation of oxygen vacancies on the (110) planes needs the least amount of energy [24]. Several theoretical studies have shown that the oxygen vacancy formation energy depends on the nature of CeO_2_ surface [25, 26]. It has also been well-established that the specific surface area of solid catalysts is a key factor in determining their overall catalytic activity in a wide number of catalytic reactions [3, 4, 27]. Therefore, it is necessary to maximize the dispersion of the nanoparticles using high-surface-area supports, such as micro- and mesoporous materials [1, 28, 29].

In the present work, a set of CeO_2_ samples with different topological and textural properties (CeO_2_ nanocubes, CeO_2_ nanorods, mesoporous CeO_2_, and CeO_2_ prepared by solution combustion synthesis) has been prepared to investigate the shape-dependency activity of ceria toward CO oxidation, a probe reaction for more complex oxidation processes. Then, the physico-chemical features of the prepared materials have been investigated using complementary techniques.

## Methods

### Synthesis of Samples

CeO_2_ nanoparticles, denoted further as “Ce-NC” and “Ce-NR” for nanocubes and nanorods, respectively, were prepared via hydrothermal procedure, using sodium hydroxide as the precipitating agent, as we described elsewhere [30]. Mesoporous CeO_2_, denoted as “Ce-MES,” was synthesized via nanocasting procedure using the SBA-15 silica template [30]. Lastly, a comparative CeO_2_ sample was prepared via solution combustion synthesis (SCS) and further referred to as “Ce-SCS.” A typical synthesis involved dissolving 3.8 g of Ce(NO_3_)_3_·6H_2_O and 1.8 g of urea in 60 ml of deionized water at room temperature. The homogeneous solution was put into a ceramic crucible and then placed in a furnace at 650 °C for 20 min.

### Catalyst Characterization

Powder X-ray diffraction (XRD) patterns were acquired on an X’Pert Philips PW3040 diffractometer using Cu Kα radiation. The XRD spectra were recorded in the 2*θ* range of 20°–70° with 0.02° step size and a time per step of 0.2 s. The diffraction peaks were identified using powder diffraction files by the International Centre of Diffraction Data (ICDD). The average crystallite size was determined using Scherrer’s equation, *D* = 0.9*λ*/*b*cos*θ*, where *λ* is the wavelength of the Cu K*α* radiation, *b* is the full width at half maximum (FWHM) in radians, 0.9 is the shape factor for spherical particles, and *θ* is the angle of diffraction peaks.

BET-specific surface areas (*S*_BET_) and total pore volumes (*V*_p_) were measured through N_2_ physisorption at −196 °C on a Micrometrics ASAP 2020 instrument. The samples were previously outgassed at 200 °C for 2 h to remove water and other atmospheric contaminants. The specific surface area of the samples was calculated using the BET method.

Sample morphology was investigated through a field emission scanning electron microscope (FESEM; Zeiss MERLIN, Gemini-II column) and transmission electron microscopy (TEM; Jeol JEM 3010 operating at 200 kV).

Redox properties of the catalysts were investigated by means of X-ray photoelectron spectroscopy (XPS). The measurements were carried out on XPS PHI 5000 Versa probe apparatus using a band-pass energy of 187.85 eV, a 45° takeoff angle, and a 100.0-μm-diameter X-ray spot size. Curve fits were performed by means of Multipack 9.0 software.

Reducibility of the catalysts was determined by H_2_-TPR measurements. Before the analyses, 50 mg of catalyst was pre-treated under air flow (40 cm^3^ min^−1^) at 150 °C for 1 h, to remove water and other atmospheric contaminants, and then cooled to 25 °C with Ar. The analysis was conducted by heating the sample up to 950 °C at a constant rate of 5 °C min^−1^ under an Ar flow (4.95 % molar H_2_ in Ar). H_2_ consumption was recorded using a thermal conductivity detector (TCD).

### Catalytic Activity Tests

Catalytic tests for CO oxidation were carried out via a classical temperature-programmed combustion (TPC). The tests were conducted in a fixed-bed quartz reactor (4-mm inner diameter, U-tube) heated by a vertical tubular furnace. The reactor bed comprised of 0.1 g of powder catalyst. A K-type thermocouple was placed at the inlet of the reactor, in such a way that the tip was as close as possible to the catalytic bed. During the reaction, a 50 cm^3^ min^−1^ gas containing 1000 ppm of CO and 50 %-vol of air in N_2_ was continuously fed into the reactor, while the temperature of the furnace increased at a rate of 5 °C min^−1^ until complete CO conversion was reached. The CO and CO_2_ concentrations at the outlet of the reactor were measured by NDIR analyzers (ABB Uras 14). Temperatures at which 10, 50, and 90 % of CO was converted were taken as indices of the catalytic activity. The tests were conducted for 2 cycles to evaluate the stability of the catalysts.

## Results and Discussion

### Textural and Redox Properties of the Catalysts

**Fig. 1:**
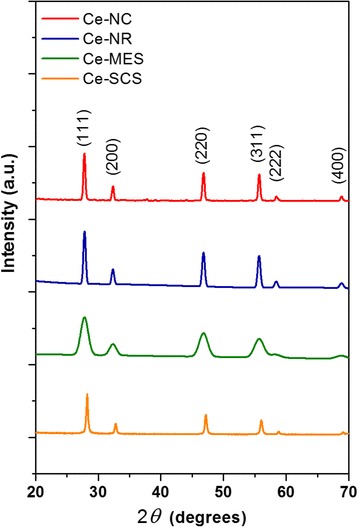
XRD patterns of the catalysts. Adapted from ref. [30]

Some main textural properties of the catalysts, obtained from N_2_ physisorption at −196 °C and X-ray diffraction analyses, are summarized in Table 1. Figure 1 shows the XRD diffractograms of the prepared catalysts. All samples exhibit similar patterns referring to cubic fluorite structure, marked by the existence of (111), (200), (220), (311), (222), and (400) planes [31]. The relative amount of the (110), (100), and (111) planes have been estimated by means of the XRD peak intensity ratios. As shown in Table 1, the highest (200)/(111) and (220)/(111) values appear for the Ce-NC sample thus showing the richest population of highly reactive (100) and (110) exposed planes. On the other hand, both the Ce-MES and Ce-SCS samples exhibit the highest amount of (111) surfaces.

**Table 1:** Textural properties of the catalysts, as obtained from N_2_ physisorption at −196 °C and X-ray diffraction analysis [30]

Catalyst	*S*_BET_	*V*_p_	Particle size	Intensity ratio^a^	Intensity ratio^a^
	(m^2^ g^−1^)	(cm^3^ g^−1^)	(nm)	(200)/(111)	(220)/(111)
Ce-NC	4	0.01	54	0.33	0.75
Ce-NR	4	0.01	43	0.32	0.71
Ce-MES	75	0.15	5	0.28	0.46
Ce-SCS	69	0.04	35	0.27	0.49

^a^XRD peak intensity ratios

However, it is worth noting that peak intensities of Ce-MES are rather broad and low. This is most likely due to nanocrystalline ceria frameworks in the sample that constitute mesoporous walls [32].

Although it is rather difficult to evaluate the average particle size through Scherrer’s formula for morphologies that differ from spherical geometry, an evaluation was conducted to corroborate the TEM results. Scherrer’s equation was employed to estimate the crystallite sizes of the catalysts: Ce-NC, Ce-NR, and Ce-SCS samples possess similar dimensions; the sizes fall in the range of 30–50 nm, whereas the mesostructured sample (Ce-MES) has the smallest particle dimension (5 nm).

The BET surface areas, obtained from N_2_ physisorption measurement at −196 °C, are very low for both the Ce-NC and Ce-NR samples (4 m^2^ g^−1^), thus suggesting a small amount of interparticle voids. In contrast, Ce-MES and Ce-SCS have much higher surface areas than their nanostructured counterparts (75 and 69 m^2^ g^−1^, respectively). Indeed, particles in the latter samples are arranged in a network that allows a creation of pores. Ce-MES has higher pore volume than Ce-SCS (0.15 and 0.04 cm^3^ g^−1^ for Ce-MES and Ce-SCS, respectively). This supports the fact that pores in Ce-MES are present in mesoscale and that those in Ce-SCS most likely exist in nanoscale.

**Fig. 2:**
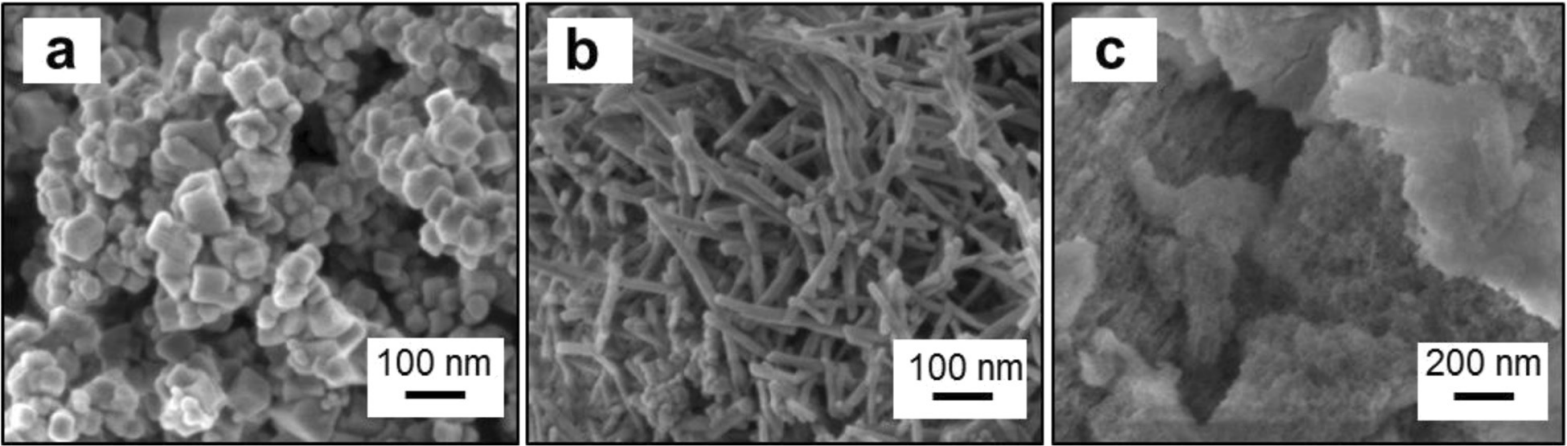
FESEM images of the **a** Ce-NC, **b** Ce-NR, and **c** Ce-MES samples

**Fig. 3:**
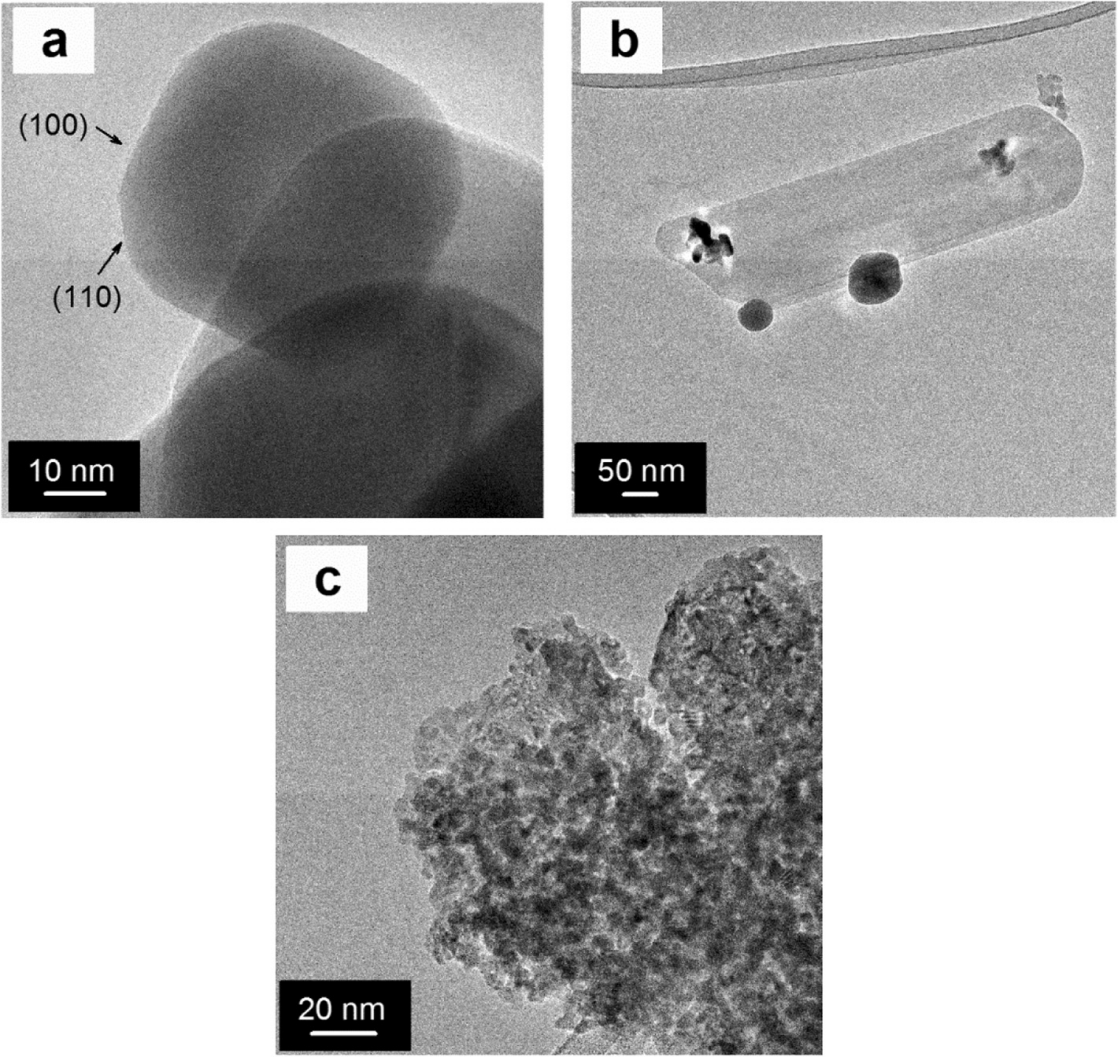
HRTEM images of the **a** Ce-NC, **b** Ce-NR, and **c** Ce-MES samples. Adapted from ref. [30]

Electron microscopy techniques were applied to find out the morphological properties of the catalysts. FESEM images of Ce-NC and Ce-NR are shown in Fig. 2a, b. The images show the morphology of nanostructured CeO_2_ (namely cubes and rods, respectively). Nanocubes are present in the size range of 50 to 200 nm, whereas nanorods can be observed, with lengths of about 300–350 nm. In contrast, Ce-MES sample (Fig. 2c) comprises of self-assembled agglomerates of small particles (average size about 50 nm) with interparticle voids. The morphology of Ce-SCS, as reported elsewhere [30, 33, 34], exhibits typically foamy structures formed by agglomerates of small particles. The representative TEM image of Ce-NC, as seen in Fig. 3a, shows the morphology of truncated nanocubes with abundant (100) surfaces and additional (110) surfaces at their truncated corners. The truncated shape is a result of the reduced surface energy; sufficient energy would give rise to a well-defined cubic structure [35]. Figure 3b shows the TEM image of Ce-NR, displaying nanorods with a width of approximately 70 nm and a length of about 350 nm. A more profound analysis with TEM, not reported here for the sake of brevity, shows many (111) planes on Ce-NR. Such a surface has higher stability and possesses lower oxidation activity than the (100) and (110) surfaces [36, 37]. The Ce-MES sample exhibits uniform particles whose sizes range from 4 to 10 nm. These particles are interconnected with each other creating a three-dimensional mesoporous network. Deeper observation with TEM discovered the exposure of many (111) lattice planes [30]. TEM analysis of Ce-SCS, as reported in our previous work [30], showed non-uniform polycrystalline ceria nanoparticles whose dimension ranges from 20 to 40 nm.

**Fig. 4:**
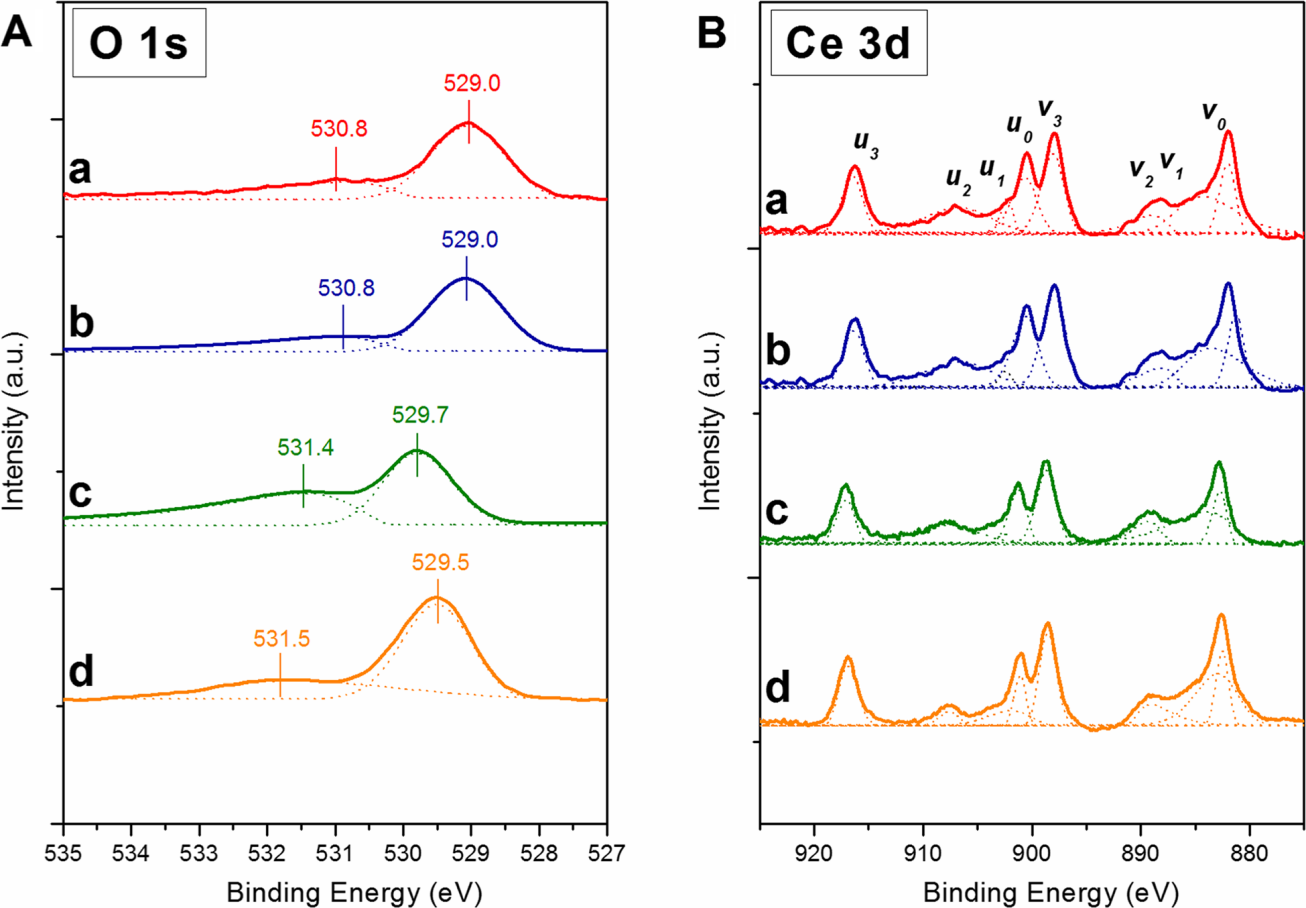
XPS spectra of the *a* Ce-NC, *b* Ce-NR, *c* Ce-MES, and *d* Ce-SCS samples in the O (1*s*) (**a**) and Ce (3*d*) core level regions (**b**). Adapted from ref. [30]

Redox properties of the catalysts were investigated by means of X-ray photoelectron spectroscopy. Figure 4a reports the XPS spectra of all samples in the O 1*s* core level. Two peaks are generally observed in the spectra: the first peak in the higher binding energy range (529.0–529.7 eV) refers to weakly adsorbed oxygen at the surface of the catalyst (O_α_ species) and the second peak in the lower binding energy range (530.8–531.5 eV) signifies the presence of oxygen in the crystal lattice of the catalyst [29, 32, 38, 39]. In general, the Ce-NC and Ce-NR samples possess a significantly low population of chemisorbed oxygen species due to their low O_α_-to-O_β_ ratios (Table 2). Richer lattice oxygen species translate as easier oxidation reaction via a Mars-van Krevelen mechanism, which is known to govern the reaction of CO oxidation [1, 5, 8, 9]. Figure 4b reports the XPS spectra of all samples in the Ce 3*d* core level. The doublets (*v*_0_,*u*_0_), (*v*_2_,*u*_2_), and (*v*_3_,*u*_3_) were attributed to the Ce^4+^ state while the doublet (*v*_1_,*u*_1_) was attributed to the Ce^3+^ state [40]. Relative abundances of Ce^3+^ and Ce^4+^ (in atomic percentage, %) estimated from deconvoluted peaks of the Ce 3*d* spectra are summarized in Table 2. Among all the samples, Ce-SCS possesses the highest concentration of Ce^3+^, most likely owing to the highly exotermic reaction during the synthesis (≥600 °C). Ce-SCS sample is also the most reducible catalyst among the series, mainly due to rich chemisorbed oxygen content on the catalyst surface.

**Table 2:** Results of XPS spectra curve fittings on the O 1*s* and Ce 3*d* core level [30]

Sample	O_α_/O_β_	Ce^3+^ (*u*_1_ + *v*_1_) (%-atom)
Ce-NC	0.04	27.6
Ce-NR	0.04	25.5
Ce-MES	1.03	25.5
Ce-SCS	0.62	36.1

### Reducibility of Catalysts

**Fig. 5:**
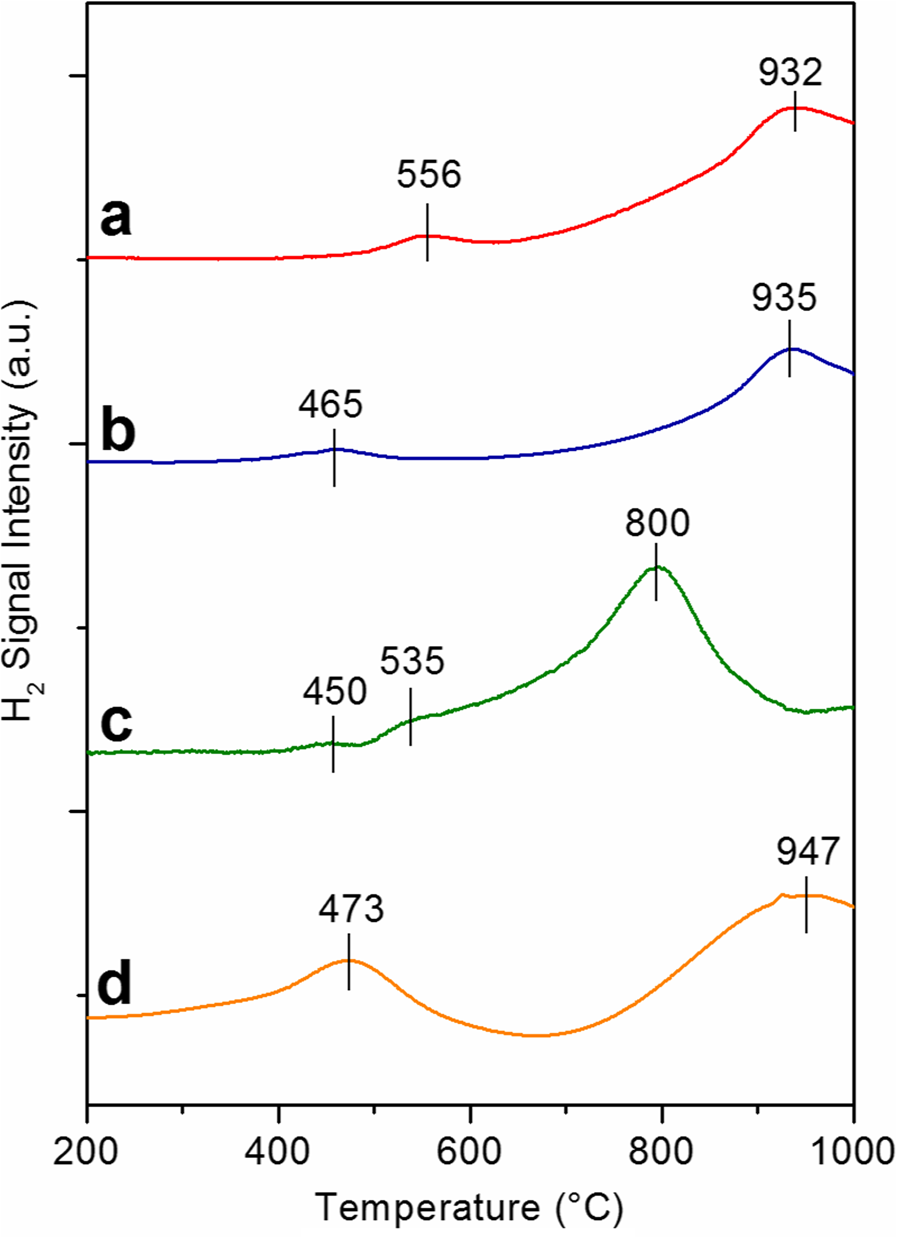
H_2_-TPR profiles of the **a** Ce-NC, **b** Ce-NR, **c** Ce-MES, and **d** Ce-SCS samples. Adapted from ref. [30]

Analyses with H_2_-TPR were carried out to understand the reduction behavior of the catalysts. Figure 5 shows the H_2_-TPR results of all samples. A typical reduction curve for cerium dioxide comprises of two peaks: (1) low-temperature reduction peak in the range of 400–500 °C that characterizes the presence of chemisorbed oxygen and (2) high-temperature reduction peak in the range of 700–1000 °C that marks the slow release of lattice oxygen (“bulk reduction”). The low-temperature reduction peak for Ce-NC (curve a) is the highest among all catalysts (556 °C), confirming the low reducibility of the catalyst owing to the scarcity of surface-capping oxygen. A similar trend is also observed for Ce-NR (curve b), although the first peak appears at a lower temperature (465 °C). The analysis with Ce-MES sample (curve c) shows two reduction peaks centered at 450 and 535 °C, due to the surface reduction of small CeO_2_ crystals, whereas the bulk reduction only occurs at 800 °C. Ce-SCS sample conforms the similar reduction trend; however, the second peak appears at the highest temperature (950 °C). This is most probably because of the low O_α_-to-O_β_ ratio.

### Catalytic Activity Tests

A series of tests with a classical TPC approach were carried out to investigate the role of structural and electronic properties on the activity of catalysts during CO oxidation reaction. A typical test was conducted according to this sequence: (1) reactor heat-up from ambient to 150 °C with air to remove any possible impurities; (2) introduction of the mixed gas (1000 ppm of CO and 50 %-vol of air in N_2_) to the reactor; and (3) second reactor heat-up from 150 °C to the temperature at which 100 % CO conversion is reached, (TOS = 2 h at 100 % conversion).

**Fig. 6:**
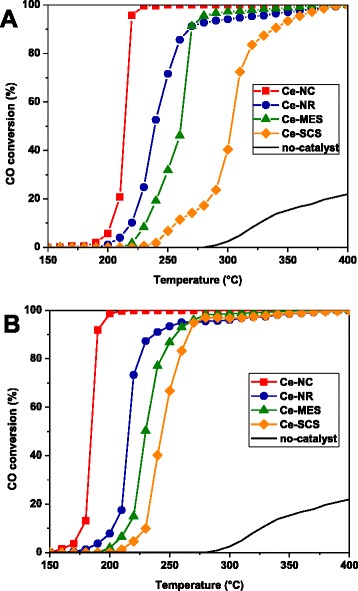
CO to CO_2_ conversion versus temperature over the “fresh” and “used” catalysts (**a** and **b**, respectively)

Figure 6a shows CO conversions as a function of reactor bed temperature over the fresh catalysts. In general, a complete CO oxidation is attainable between 150 and 400 °C. Table 3 summarizes the temperatures at which 10, 50, and 90 % of CO are converted. It appears that the performance of the catalysts follow this order: Ce-NC > Ce-NR > Ce-MES > Ce-SCS; the lowest oxidation temperature was obtained for Ce-NC (T_50%_ = 214 °C) due to the larger number of reactive (100) and (110) surfaces. This suggests the beneficial effect of well-defined nanostructures by which some active surfaces are possibly generated. The nanocubic structures in the Ce-NC sample allow highly reactive (100) and (110) low-index surfaces to be exploited, due to their low coordination numbers on the surface and better redox properties [30, 41], as revealed by XPS and H_2_-TPR analysis. However, the Ce-SCS and Ce-MES samples, despite their higher surface areas, exhibit lower oxidation reactivity due to the abundance of stable (111) surfaces that constitute the catalysts’ structures. This may be inferred that CO oxidation reaction can be structure sensitive over ceria materials. These findings are in agreement with previous work [29, 30, 42–44], according to which ceria-based catalysts, exhibiting highly reactive planes, show promising catalytic activities for diesel soot combustion despite their low textural properties.

**Table 3:** CO oxidation activity results over ceria-based catalysts

Sample	1st cycle			2nd cycle		
	T_10%_	T_50%_	T_90%_	T_10%_	T_50%_	T_90%_
Ce-NC	203	214	219	177	185	190
Ce-NR	220	239	268	203	216	237
Ce-MES	232	260	270	214	230	255
Ce-SCS	257	303	338	230	244	266

In order to evaluate the stability of the catalysts, as well as the reliability of the data, re-tests have been carried out with previously used catalysts. Figure 6b shows CO conversions as a function of temperature over the “used” catalysts. The activity order of the catalysts appears to be preserved, with Ce-NC still being the most active catalyst. Interestingly, it was found that the re-tests with used catalysts resulted in better CO oxidation activity compared to those with “fresh” catalysts. The temperature indicators showed values 10–20 °C lower than those in the tests with fresh catalysts. The exposure of CO during the first cycle might give some carbonate species (e.g., CO_3_^2−^) on the solid surface that would help improve CO oxidation. It is known that oxygen spillover at catalyst surface contributes to the CO oxidation [45], and so, an abundant presence of surface polycarbonates, stable at high temperature, would have a beneficial effect on the reactivity of ceria nanocatalysts, as already observed by FT-IR analysis over ceria-zirconia catalysts [46].

## Conclusions

As a whole, it has been observed that the catalytic performance for CO oxidation over CeO_2_ nanocatalysts mainly depends on the presence of highly reactive (100) and (110) surfaces, thus confirming the structure sensitivity for this prototypical reaction. The best results, in terms of CO oxidation, were achieved for the Ce-NC catalyst, due to the abundance of coordinative unsaturated atomic sites in the (100) and (110) exposed surfaces of the CeO_2_-truncated nanocubes. Moreover, this nanostructured catalyst has shown the lowest reducibility (*vide* H_2_-TPR data), thus validating the structure dependency for the CO oxidation reaction. On the other hand, worse CO conversion values were obtained for the high-surface-area catalysts (CeO_2_-MES and CeO_2_-SCS), thus showing the key role played by the structural properties of ceria. The least active catalyst was the CeO_2_-SCS, exhibiting the highest amount of stable (111) planes.

## References

[1] Trovarelli A, Fornasiero P (2013). Catalysis by ceria and related materials.

[2] Heck RM, Farrauto RJ, Gulati ST (2006). Catalytic air pollution control: commercial technology.

[3] Ertl G, Knözinger H, Schüth F, Weitkamp J (2008). Handbook of heterogeneous catalysis.

[4] Duprez D, Cavani F (2014). Handbook of advanced methods and processes in oxidation catalysis.

[5] Wu Z, Li M, Overbury SH (2012). On the structure dependence of CO oxidation over CeO_2_ nanocrystals with well-defined surface planes. J Catal..

[6] Russo N, Fino D, Saracco G, Specchia V (2006). Supported gold catalysts for CO oxidation. Catal Today..

[7] Freund HJ, Meijer G, Scheffler M, Schlögl R, Wolf M (2011). CO oxidation as a prototypical reaction for heterogeneous processes. Angew Chem Int Ed..

[8] Royer S, Duprez D (2011). Catalytic oxidation of carbon monoxide over transition metal oxides. ChemCatChem..

[9] Aneggi E, Llorca J, Boaro M, Trovarelli A (2005). Surface-structure sensitivity of CO oxidation over polycrystalline ceria powders. J Catal..

[10] Tana, Zhang M, Li J, Li H, Li Y, Shen W (2009). Morphology-dependent redox and catalytic properties of CeO_2_ nanostructures: nanowires, nanorods and nanoparticles. Catal Today.

[11] Liu X, Zhou K, Wang L, Wang B, Li Y (2009). Oxygen vacancy clusters promoting reducibility and activity of ceria nanorods. J Am Chem Soc..

[12] Yan L, Yu R, Chen J, Xing X (2008). Template-free hydrothermal synthesis of CeO_2_ nano-octahedrons and nanorods: investigation of the morphology evolution. Cryst Growth Des..

[13] Guan Y, Hensen EJM, Liu Y, Zhang H, Feng Z, Li C (2010). Template-free synthesis of sphere, rod and prism morphologies of CeO_2_ oxidation catalysts. Catal Lett..

[14] Lin KS, Chowdhury S (2010). Synthesis, characterization, and application of 1-D cerium oxide nanomaterials: a review. Int J Mol Sci..

[15] Han WQ, Wen W, Hanson JC, Teng XW, Marinkovic N, Rodriguez JA (2009). One-dimensional ceria as catalyst for the low-temperature water-gas shift reaction. J. Phys. Chem. C.

[16] Sau TK, Rogach AL (2012). Complex-shaped metal nanoparticles: bottom-up syntheses and applications.

[17] Wang ZL, Feng X (2003). Polyhedral shapes of CeO_2_ nanoparticles. J Phys Chem B..

[18] Teng B, Jiang S, Guo X, Yuan J, Luo M (2009). A density functional theory study of CO oxidation on CeO_2_ (110) surface. Acta Chim Sinica..

[19] Nolan M (2009). Molecular adsorption on the doped (110) ceria surface. J Phys Chem C..

[20] Muller C, Paulus B, Hermansson K (2009). Ab initio calculations of CO physisorption on ceria (111). Surf Sci..

[21] Huang M, Fabris S (2008). CO adsorption and oxidation on ceria surfaces from DFT + U calculations. J Phys Chem C..

[22] Scanlon DO, Galea NM, Morgan BJ, Watson GW (2009). Reactivity on the (110) surface of ceria: a GGA + U study of surface reduction and the adsorption of CO and NO_2_. J Phys Chem C..

[23] Alam MK, Ahmed F, Nakamura K, Suzuki A, Sahnoun R, Tsuboi H, Koyama M, Hatakeyama N, Endou A, Takaba H, Del Carpio CA, Kubo M, Miyamoto A (2009). Study of carbon monoxide oxidation on CeO_2_ (111) using ultra accelerated quantum chemical molecular dynamics. J Phys Chem C..

[24] Cheng Z, Sherman BJ, Lo CS (2013). Carbon dioxide activation and dissociation on ceria (110): a density functional theory study. J Chem Phys..

[25] Nolan M, Fearon JE, Watson GW (2006). Oxygen vacancy formation and migration in ceria. Solid State Ionics..

[26] Nolan M, Parker SC, Watson GW (2005). The electronic structure of oxygen vacancy defects at the low index surfaces of ceria. Surf Sci..

[27] Liu Y, Wen C, Guo Y, Lu G, Wang Y (2010). Effects of surface area and oxygen vacancies on ceria in CO oxidation: differences and relationships. J Mol Catal A..

[28] Cejka J, Corma A, Zones S (2010). Zeolites and catalysis: synthesis, reactions and applications.

[29] Piumetti M, Bensaid S, Russo N, Fino D (2016). Investigations into nanostructured ceria–zirconia catalysts for soot combustion. Appl Catal B..

[30] Piumetti M, Bensaid S, Russo N, Fino D (2015). Nanostructured ceria-based catalysts for soot combustion: investigations on the surface sensitivity. Appl Catal B..

[31] Laha SC, Ryoo R (2003) Synthesis of thermally stable mesoporous cerium oxide with nanocrystalline frameworks using mesoporous silica templates. Chem Commun 17:2138-2139 10.1039/b305524h 13678169

[32] Ji P, Zhang J, Chen F, Anpo M (2008). Ordered mesoporous CeO_2_ synthesized by nanocasting from cubic Ia3d mesoporous MCM-48 silica: formation, characterization and photocatalytic activity. J Phys Chem C..

[33] Kumar PA, Tanwar MD, Bensaid S, Russo N, Fino D (2012). Soot combustion improvement in diesel particulate filters catalyzed with ceria nanofibers. Chem Eng J..

[34] Miceli P, Bensaid S, Russo N, Fino D (2015). Effect of the morphological and surface properties of CeO2-based catalysts on the soot oxidation activity. Chem. Eng. J.

[35] Agarwal S, Lefferts L, Mojet BL (2013). Ceria nanocatalysts: shape dependent reactivity and formation of OH. ChemCatChem..

[36] Yang Z, Woo TK, Baudin M, Hermansson K (2004). Atomic and electronic structure of unreduced and reduced CeO2 surfaces: a first-principles study. J Chem Phys..

[37] Agarwal S, Lefferts L, Mojet BL, Ligthart DAJM, Hensen EJM, Mitchell DRG, Erasmus WJ, Anderson BG, Olivier EJ, Neethling JH, Datye AK (2013). Exposed surfaces on shape‐controlled ceria nanoparticles revealed through AC‐TEM and water–gas shift reactivity. ChemSusChem..

[38] Zhang G, Shen Z, Liu M, Guo C, Sun P, Yuan Z, Li B, Ding D, Chen T (2006). Synthesis and characterization of mesoporous ceria with hierarchical nanoarchitecture controlled by amino acids. J Phys Chem B..

[39] Sinha AK, Suzuki K (2005). Preparation and characterization of novel mesoporous ceria-titania. J Phys Chem B..

[40] Brezesinski T, Erpen C, Iimura K, Smarsly B (2005). Mesostructured crystalline ceria with a bimodal pore system using block copolymers and ionic liquids as rational templates. Chem Mater..

[41] Paier J, Penschke C, Sauer J (2013). Oxygen defects and surface chemistry of ceria: quantum chemical studies compared to experiment. Chem Rev..

[42] Fino D, Bensaid S, Piumetti M, Russo N (2016). A review on the catalytic combustion of soot in diesel particulate filters for automotive applications: from powder catalysts to structured reactors. Appl. Catal. A.

[43] Miceli P, Bensaid S, Russo N, Fino D (2014). CeO_2_-based catalysts with engineered morphologies for soot oxidation to enhance soot-catalyst contact. Nanoscale Res. Lett..

[44] Bensaid S, Russo N, Fino D (2013). CeO_2_ catalysts with fibrous morphology for soot oxidation: the importance of the soot-catalyst contact conditions. Catal. Today.

[45] Thomas JM, Thomas WJ (2015) Principles and practice of heterogeneous catalysis (2n Edition). Wiley-VCH Verlag, Weinheim, Germany 113-126

[46] Piumetti M, Bensaid S, Fino D, Russo N (2016) Ceria-zirconia nanocatalysts for CO oxidation: study on surface properties and reactivity. Appl Catal B http://dx.doi.org/10.1016/j.apcatb.2016.02.023

